# Integrated Water Resource Management under Ecosystem Services Approach—The Chimulala Micro-watershed, Peru

**DOI:** 10.1007/s00267-024-02092-z

**Published:** 2024-12-06

**Authors:** Luisa Fernanda Cifuentes-Herrera, Luz Piedad Romero-Duque, Oscar Eduardo Angulo Núñez, Jenny Maritza Trilleras

**Affiliations:** 1 Pontificia Universidad Católica de Perú, Av. Universitaria 1801, Lima, 15088 Perú; 2 Universidad de Ciencias Aplicadas y Ambientales, Calle 222 No. 55-37, Bogotá, Colombia

**Keywords:** Stakeholders, Sociocultural valuation, Perception value, Orientation value, Change value and water management

## Abstract

This study evaluates the sociocultural valuation of ecosystem services (ES) within the Chimulala micro-watershed, Peru, to inform Integrated Water Resource Management (IWRM). Using surveys and focus groups, we gathered data from 35 stakeholders (11 institutional and 24 local actors) to assess perceptions, orientations, and anticipated changes regarding 15 identified ES. A land cover map was developed to support an expert-led ES assessment, categorizing capacity levels across different land types. Results showed unanimous agreement on the vital importance of the water supply service, with local actors identifying and valuing a broader array of ES than institutional counterparts. Multiple correspondence analysis highlighted differences in ES recognition between stakeholder groups, revealing that local actors ascribed greater importance to cultural and regulatory ES, while institutional actors prioritized provisioning services. Conflicts were identified regarding responsibilities for changes in ES provision, with local communities attributing threats primarily to mining, while institutional actors cited land use changes. This research underscores the value of sociocultural ES assessments in bridging divergent perspectives and enhancing the participatory foundation of IWRM, ultimately aiding in the design of inclusive, resilient water management policies.

## Introduction

Water is fundamental to human development, serving not only to meet essential and spiritual needs but also supporting ecosystem functions, biodiversity conservation, and use in activities such as energy generation, crop irrigation, waste disposal, transportation, and industrial production (Gordon et al. 2015). It is a critical resource for global advancement (Martínez and Villalejo 2018). Global water demand is anticipated to grow at a rate of 1% annually, with 69% of this demand attributed to the agricultural sector, 19% to industry, and 12% to domestic use. Additionally, demand in industrial and domestic sectors is projected to increase by 20–30% by 2050 (WWAP, 2019). This demand generates conflicts arising from competing interests among water users—agriculture, industry, and conservation—and the pressures these interests place on ecosystems, which, despite their protected status, remain vulnerable to human activities (Castro-Pardo et al. 2021).

Given these issues, IWRM faces challenges in implementing inclusive and participatory approaches (Galvez and Rojas 2019). Another key problem affecting IWRM is inefficient or absent governance across various management scales—national, regional, and local—particularly in developing countries (GWP 2000; Katusiime and Schütt 2020). Policy design and implementation remain isolated and fragmented (Niasse and Cherlet 2015; Castro et al. 2016), often failing to involve all actors in decision-making or to recognize the interconnectedness among diverse water users (Niasse and Cherlet 2015 and Ncube et al 2021). This disconnect heightens competition and social conflict, exacerbated by limited water availability (Amprako 2016; Mekonnen and Hoekstra 2016). Such management practices continue to fuel conflicts over water use and governance across local, regional, and national levels, as well as between rural and urban areas (Hommes and Boelens 2017).

To address these challenges, IWRM advocates for decentralized and collective water management (González 2017). However, in practice, productive uses are often prioritized (Niasse and Cherlet 2015; Castro et al. 2016), with economic values of water given precedence over its sacred, cultural, historical, and political significance, all of which contribute to human well-being (Devkota et al. 2018). Niasse and Charlet (2015) and Grizzetti et al. (2016) propose integrating the ecosystem services (ES) approach with IWRM to enhance management frameworks. This integration, the authors argue, brings to light the hidden benefits of water bodies for human health and supports multifunctionality and sustainability in water management.

From an ES perspective, the integration of biophysical, economic, and sociocultural valuation domains is essential for identifying optimal management alternatives (Martín-López et al. 2012; Gómez-Baggethun et al. 2014; Pascual et al. 2010). Within the interaction framework between IWRM and the ES approach, biophysical and economic valuation domains, along with their intersections, are the most frequently addressed (Grizzeti et al. 2016 and Ekka et al. 2020). The sociocultural valuation of ES is defined as: “*the importance that people, as individuals or as a group, give to ecosystem services*” (Scholte et al. 2015). Despite its significance in tackling management challenges, sociocultural assessment remains underexplored, and its evaluation and integration for fostering local participation are still unclear (Chan et al. 2012a; Chan et al. 2012b; Martín-López et al. 2012; Ramos et al. 2018; Brauman 2015; Castro et al. 2016). In this regard, the Intergovernmental Science-Policy Platform on Biodiversity and Ecosystem Services (IPBES) underscores the importance of co-constructing integrated knowledge to analyze and manage human-nature relationships, thereby emphasizing sociocultural value as critical for decision-making (Díaz et al. 2015).

Gevara (2019), notes that IWRM in Peru often fails to incorporate local perspectives and priorities. In Peru, Payments for Environmental Services (PES)—known locally as Mechanisms for Rewarding Ecosystem Services (MRSE)—are being implemented. These instruments mobilize economic resources to sustain, conserve, and restore ecosystems by channeling funds from downstream users through a percentage of the water tariff to upstream providers, thereby protecting water resources and their basins (Ley No. 30215, 2014; Angulo, n.d). One priority basin under various PES initiatives is the Jequetepeque River basin, located on the western slopes of the Andes in northern Peru (Quintero-Pareja 2015). Within this basin lies the Gallito Ciego Dam, which regulates water flow and has facilitated extensive agricultural and livestock activities downstream. However, shifts in land cover from natural vegetation to predominantly agricultural and livestock uses in the middle and upper basin have led to conflicts related to sediment load dynamics, including dam clogging, nutrient loss from soils in the lower basin, and potential impacts on the coastline (ANA 2015; Tavares 2015).

The Jequetepeque River basin is currently included in the project Conservation and Sustainable Use of the High Andean Ecosystems of Peru through PES for the Alleviation of Rural Poverty and Social Inclusion, MERESE-FIDA. This initiative aims to conserve high Andean ecosystems to ensure the provision of ecosystem services, with a focus on biodiversity and water-related services (MINAM and FIDA 2018). Within this basin, the MERESE-FIDA project designated the Chimulala micro-watershed (located in the Pallac River sub-basin, a primary tributary of the Jequetepeque River with an approximate flow of 0.80 m³/s) as a control site in its peer micro-watershed analysis for monitoring project outcomes. Consequently, the inhabitants of the Chimulala micro-watershed have not participated in awareness-raising processes and thus lack predispositions regarding ES. The Chimulala micro-watershed, situated in the San Miguel district, is critical to the MERESE-FIDA project because it hosts the most extensive native forest coverage (MINAM and FIDA 2018) and has the largest population, which is predominantly rural and engaged primarily in agriculture and livestock activities.

We conducted a sociocultural valuation of the ES provided by the Chimulala micro-watershed’s ecosystems, focusing on the values of perception, orientation, and change across various actors. This approach aimed to understand the perceptions, preferences, and motivations that influence or alter ES provision and delivery. Given that water provision is closely tied to ecosystem conservation and related ES, we propose that sociocultural valuation of ES can significantly inform decision-making within IWRM. This research is especially pertinent as a complementary measure to PES, where prioritization is typically based on socioeconomic considerations and supply demands in the lower basin for predominantly productive uses (Stern and Echavarría 2013).

## Study Site

**Fig. 1:**
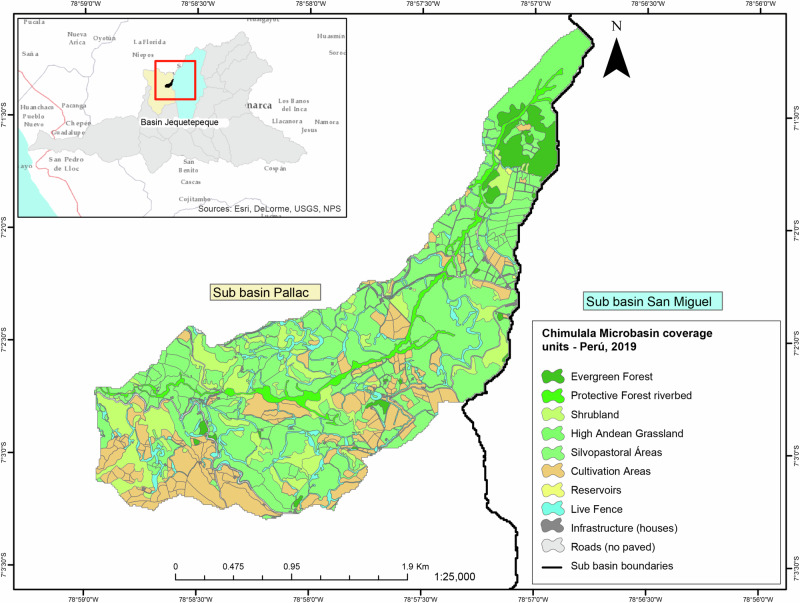
Location of the Chimulala micro-watershed within the Jequetepeque Basin (Cajamarca, Peru)

The Chimulala micro-watershed is located within the Pallac River sub-basin of the Jequetepeque River (07°04’ S; 78°96’ W), covering an area of 5.48 km² and ranging in elevation from 2774 to 3760 m above sea level (masl) (Fig. 1). The rainfall pattern is divided into a dry season (June–August) and a wet season (January-March), with transitional months experiencing water deficits, particularly in July (MINAM and FIDA, 2018). The Pallac River sub-basin exhibits diverse land cover, from Tropical Lower Montane Very Humid Forest to Tropical Desert Scrub, with Tropical Montane Dry Forest occupying the largest portion (26%). The micro-watershed lies in an area highly susceptible to erosion (MINAM and FIDA, 2018), and significant ecosystem transformations have taken place, notably deforestation and subsistence cropping (Huaman, 2016; López and Giron 2007). The peasant communities, La Arteza (394 inhabitants), Mutish (126 inhabitants), and Rodeopampa (215 inhabitants), rely entirely on the basin for their various needs (INEI 2017).

## Methodology

To assess the capacity of the Chimulala micro-watershed to provide ES, we conducted an expert consultation. The selection criteria for experts included: having conducted research on the biophysical valuation of ES with at least one scientific publication; possessing knowledge of and research experience in Peru, specifically in the Cajamarca region. Given that most research in the area centers on economic valuation, we also selected experts with internationally published work and knowledge of high Andean ecosystems. Lastly, the likelihood of response was considered, as initial contact was made via email, and prior interactions increased the probability of a favorable response. Based on these criteria, we consulted with 11 experts.

To accomplish this, we created a land cover map at a 1:5000 scale, with a minimum mappable area of 0.064 ha. Following Salitchev (1979), we performed an unsupervised digital classification with ten spectral classes. We use a 2016 Landsat 8 multispectral satellite image with a 30 m spatial resolution provided by the MERSE project. Using the resulting raster and the 2.5 m from ArcGis 10.2.2 SPOT image (World Imagery base map), we generated a thematic map (vector model and 1:2000 scale) (Fig. 1). Finally, we validated and adjusted the map base on field control point data (Li et al. 2006). After completing the map, we created a matrix with the Y-axis representing the list of identified cover types and the X-axis representing ES, as classified by TEEB (2010). Experts then assessed the capacity of micro-watershed land cover to provide ES using a symmetric Likert scale (Joshi et al. 2015): 0 = no relevant capacity, 1 = very low, 2 = low, 3 = medium, 4 = high, and 5 = very high. We conducted a frequency analysis of responses for each ES by land cover based on these results.

To carry out the sociocultural valuation of ES (perception, orientation, and value changes), we applied the stakeholder classification from the MERESE FIDA Project, which organizes participants into institutional and local actors. This research included 35 participants: 11 institutional actors and 24 local actors. The eleven institutional actors represented public institutions with political and administrative decision-making authority over the Jequetepeque River basin, encompassing the Chimulala micro-watershed. These institutional actors held management responsibilities within the middle-upper basin (4 actors), lower basin (3 actors), and across the entire basin (4 actors).

The 24 local actors were comprised of leaders and social groups from the three previously mentioned rural communities (La Arteza, Mutish, and Rodeopampa) within the micro-watershed. These Rural Communities are legally recognized organizations with juridical status, connected by ancestral, social, economic, and cultural ties, expressed through communal land ownership (Ley No. 24656). Each Rural Community is governed by a Community Board, with the President of the Community serving as the main leader, elected by vote in a Community Assembly and responsible for governance and administration. The organizational structure also includes specialized committees addressing specific issues. The 24 local actors participating in this study represent these various specialized committees. Among the actors interviewed were deputy and municipal agents, community presidents, presidents of user boards and irrigation committees (representative organizations of water users for agricultural and livestock purposes), and leaders of state programs such as JUNTOS^1^, APAFA^2^, Vaso de Leche^3^, PRONOEI^4^, Comedor Popular^5^ and Club de Madres^6^.

Given the study’s aim to understand how different actors perceive and value ES—specifically to inform IWRM strategies in a culturally sensitive way—we conducted a sociocultural valuation of ES, as suggested in the literature (e.g. García-Nieto et al. 2015; Castro et al. 2016; Garau et al. 2020; Ekka et al. 2020 & Ncube 2021). These authors utilized various participatory techniques, including expert panels, interviews, and focus groups, to evaluate diverse ES. For example, Ekka et al. (2020) emphasized that human-induced changes in river landscapes often decrease socio-cultural ES values, impacting the biophysical environment and local communities. Similarly, studies by Ncube et al. (2021) and Garau et al. (2020) illustrate the use of participatory methodologies in culturally sensitive contexts, highlighting the significance of socio-cultural values in water management and participatory planning.

The perception value helped us determine the level of importance each stakeholder assigns to each ES. The orientation value revealed the primary motivations shaping their environmental attitudes and behaviors toward these services; we classified motivations as selfish (egoistic), unselfish (altruistic), and biospheric (De Groot and Steg 2008). Selfish motivations prioritize personal well-being, unselfish motivations prioritize the well-being of others, and biospheric motivations prioritize other species (Arias-Arévalo et al. 2017). Lastly, the change value allowed us to identify whether actors believe these services will be maintained, decrease, or increase over time, as well as the activities and individuals responsible for these changes.

Based on the ES list that experts identified as having high relevance for provision within the micro-watershed, institutional actors selected the services they deemed the micro-watershed provides. We then asked each actor to assess the values of perception, orientation, and change they ascribe to these ES. We evaluated perception using a symmetric Likert scale (Joshi et al. 2015) ranging from vitally important (VI) to unimportant (UI). Following De Groot and Steg (2008), we assessed orientation (motivation) by asking actors to select from unselfish, selfish, or biocentric motivations. Notably, several actors indicated more than one orientation, leading us to establish seven categories: (1) unselfish and selfish; (2) unselfish and biocentric; (3) selfish and unselfish; (4) selfish and biocentric; (5) biocentric; (6) unselfish; and (7) all orientations. For the change value, we inquired whether the micro-watershed’s capacity to provide ES would increase, remain stable, or decrease over time and identified the likely drivers and responsible agents for these changes.

We also conducted three focus groups with local actors, one in each of the micro-watershed’s peasant communities (La Arteza, Mutish, and Rodeopampa). In each session, we introduced the project and its objectives, provided context on the issues, and obtained signed informed consent from participants who agreed to participate in the assessment. We then explored the perceived benefits of nature by asking the following questions: (1) What benefits do you receive from nature? (2) How do you believe these benefits arise? (3) How does nature support your daily life? (4) What is the most essential thing the environment provides? and (5) What would you miss most if you moved to Lima? Responses were recorded on a numbered poster, summarized, and reviewed to ensure completeness. This process enabled us to establish a list of ES recognized by the participants, from which we gathered values for perception, orientation, and change. For the perception value, each participant received a sheet with an asymmetric Likert scale featuring color-coded expressions. For orientation, we provided sheets with representative images of each orientation category. Lastly, the change value was assessed within the focus group using the same criteria as for institutional actors.

We analyzed institutional and local actors’ perception and orientation values using frequency analysis and multiple correspondence analysis (MCA). MCA was conducted using the FactoMineR version 1.42 package (Lê et al. 2008), with confidence ellipses drawn to visualize groupings between variables through the “addEllipse” function in FactoMineR (Husson et al. 2012). All analyses were performed in R Studio v. 3.6.1 (R Core Team, 2019). This approach enabled us to distinguish variations in responses across different management levels among institutional actors and between local actors, based on demographic factors and location within the micro-watershed.

Finally, a discourse analysis was conducted following Bardin and Suárez (1986), based on focus group discussions. From the focus group transcriptions, we identified words and phrases linked to each ES group, allowing us to align services recognized by local actors with the TEEB (2010) classification. The discourse analysis also helped establish the exchange value, identify activities potentially impacting these changes, and determine those responsible for them.

## Results

### Capacity of the micro-watershed land cover to provide ecosystem services

According to the expert panel, the land cover types in the Chimulala micro-watershed display varying capacities to provide ES (Table 1). Experts assigned a high or very high capacity for cultural, habitat, and regulation ecosystem services to forested areas, while they attributed provision services—particularly for raw materials and food—to crop and silvopastoral covers. Although other natural land covers received similar evaluations, forests were highlighted for providing a broader range of ecosystem services, supplying 12 of the 17 analyzed services. The reservoir was also rated as having a very high capacity for water-related services, including provision and regulation. Conversely, anthropic land covers were rated with medium to low capacity or were not recognized as relevant for ES provision.

**Table 1:**
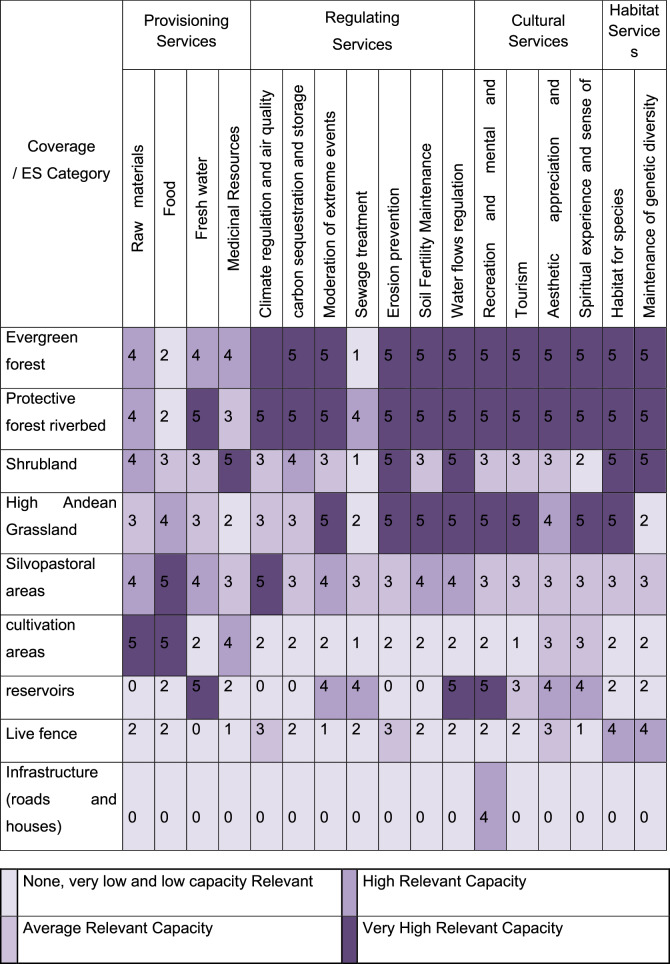
Capacity level of land cover types to provide ecosystem services in the Chimulala micro-watershed (Cajamarca, Peru)

### Sociocultural ecosystem services value

**Fig. 2:**
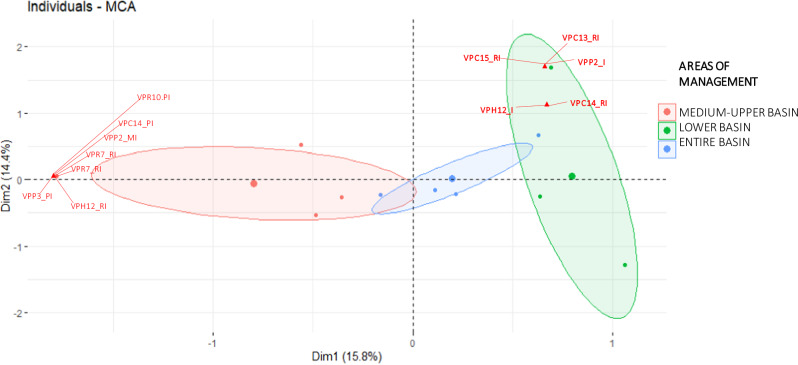
Multiple correspondence analysis for perception values assigned by local actors to ecosystem services in the Chimulala River Basin (Cajamarca, Peru). The first two dimensions represent 30% of the cumulative variance. Codes begin with “VP” for Perception value, followed by a numeric identifier for each ecosystem service (see Annex 1 for details). Subsequent letters denote categories for each value: Vital (V), Very Important (VI), Important (I), Relatively Important (R), and Unimportant (U)

**Fig. 3:**
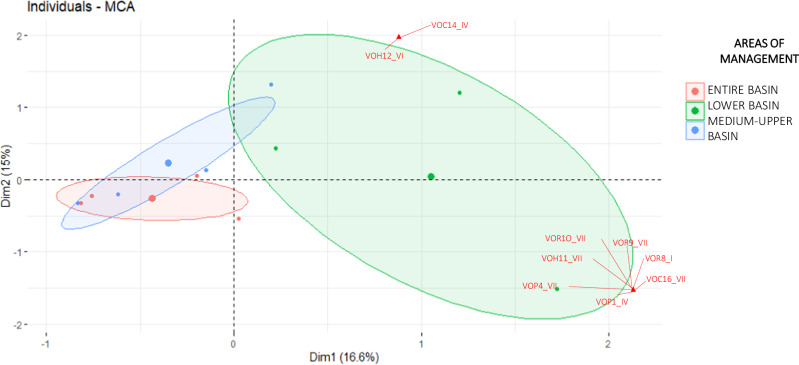
Multiple correspondence analysis for oreintation values assigned by local actors to ecosystem services in the Chimulala River Basin (Cajamarca, Peru). The first two dimensions represent 32% of the cumulative variance. Codes begin with “VO” for Orientation Value, followed by a numeric identifier for each ecosystem service (see Annex 1 for details). Subsequent letters denote categories for each value: Selfish (I), Unselfish (II), Biocentric (III), Selfish and Unselfish (IV), Selfish and Biocentric (V), Unselfish and Biocentric (VI), and All (VII)

**Fig. 4:**
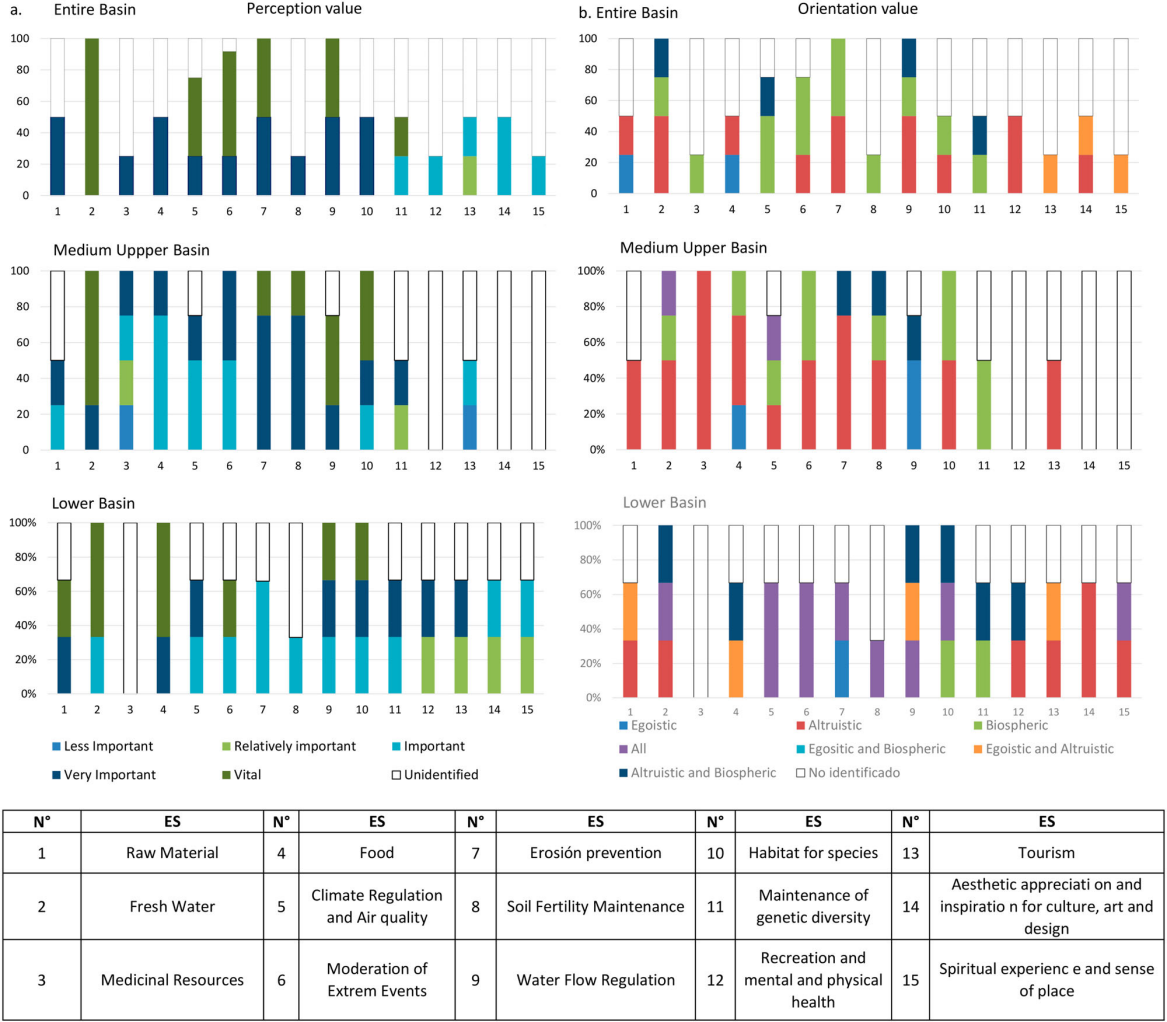
Ecosystem services’ perception (**a**) and orientation (**b**) values assigned by institutional actors in the Chimulala micro-watershed (Cajamarca, Peru)

**Fig. 5:**
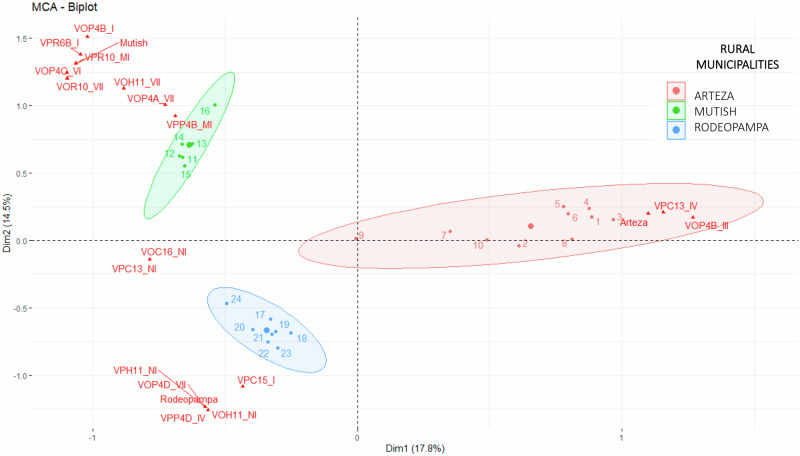
Multiple correspondence analysis for the perception and orientation values assigned by local actors to ecosystem services in the Chimulala River Basin (Cajamarca, Peru). The first two dimensions represent 32% of the accumulated variance. Coding begins with “VP” for Perception value, where categories are Vital (V), Very Important (VI), Important (I), Relatively Important (R), and Unimportant (U). For Orientation value, indicated by “VO,” categories include Selfish (I), Unselfish (II), Biocentric (III), Selfish and Unselfish (IV), Selfish and Biocentric (V), Unselfish and Biocentric (VI), and All (VII). Each code is followed by the numeric identifier for ecosystem services, as detailed in Annex 1

Some general patterns emerged in the perception, orientation, and change values of ES in the Chimulala micro-watershed. For instance, the MCA results show that institutional actors with the same management scope within the micro-watershed identified and valued similar ecosystem services (Figs. 2, 3). This alignment suggests that sociocultural assessments mirror the watershed’s management structure, where actors operate at different administrative levels: the entire basin (Local Water Authority, Basin Water Resources Council, and the Ministry of Environment’s Compensation Mechanisms Project), the middle and upper basins as a unified administrative region (Cajamarca Regional Government and San Miguel Provincial Government), and the lower basin as a separate administrative region (La Libertad Regional Government) (Fig. 4a, b). Likewise, the MCA revealed that actors from each peasant community also identified and valued ES distinctly (Fig. 5).

Both local and institutional actors recognized the regulation and provision of water-related ES as vital or very important. Local actors also identified other ES with similar importance values. However, perception, orientation, and change values for the ES of the Chimulala micro-watershed differed within and between stakeholder groups. Among institutional actors, only one of the 15 ES—water supply—was identified by all, while 90% of them recognized the regulating water flows and erosion prevention ES. There was no clear pattern for other ES, but provision and regulation services generated the most consensus. In contrast, cultural and habitat ES were the least recognized by institutional actors (Fig. 4a). Additionally, provision ES were primarily identified by men (80% of 9), while women highlighted other ES, including habitat for species, local climate and air quality regulation, soil fertility maintenance, and moderation of extreme events (100% of 2).

**Fig. 6:**
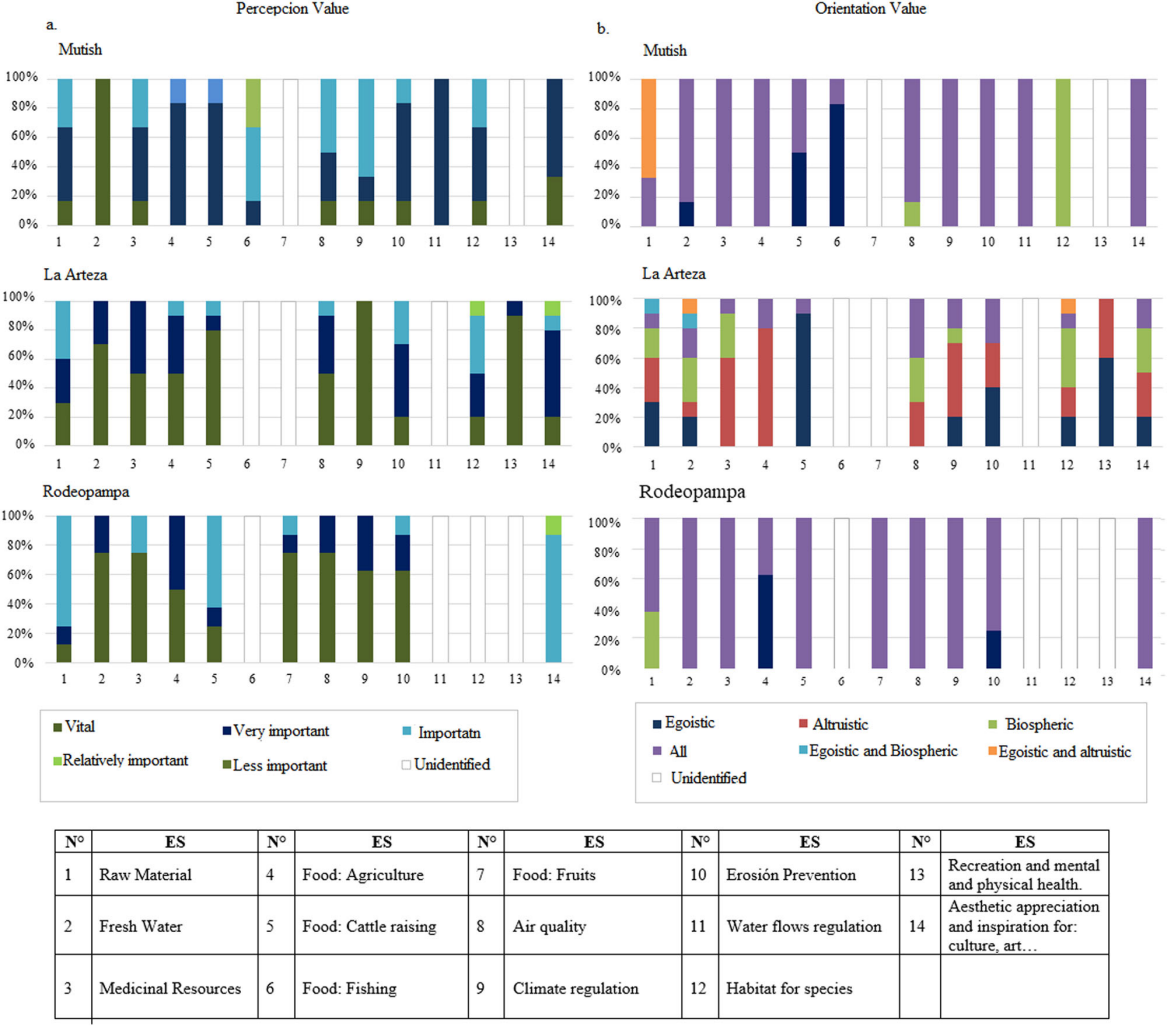
Ecosystem services’ perception (**a**) and orientation (**b**) values granted by the local actors of the Chimulala micro-watershed (Cajamarca, Peru)

Local actors identified 12 of the 15 analyzed ES. Specifically, actors from the Mutish community identified 12 ES, those from La Arteza identified 11, and those from Rodeopampa identified 9. Actors from both Mutish and La Arteza recognized the nine ES identified in Rodeopampa, which included raw materials, medicinal resources, food, water, regulation of local climate and air quality, moderation of extreme events, erosion prevention, recreation, mental and physical health, and esthetic appreciation and cultural inspiration. Additionally, actors from Mutish and La Arteza identified habitat, spiritual experiences, and a sense of place ES. Unique to Mutish was the identification of the water flow regulation ES (Fig. 6a).

Institutional actors identified multiple orientation value categories, with unselfish orientations predominant (40%), followed by categories combining unselfish with biocentric (12%), selfish/unselfish (7%), and “everyone” (12%). The “all” orientation category was most common in the lower basin (Fig. 4b). Similarly, local actors identified multiple orientation categories, with “all” predominant among actors from Mutish and Rodeopampa, while the unselfish category was most common for La Arteza actors (Fig. 6b).

**Fig. 7:**
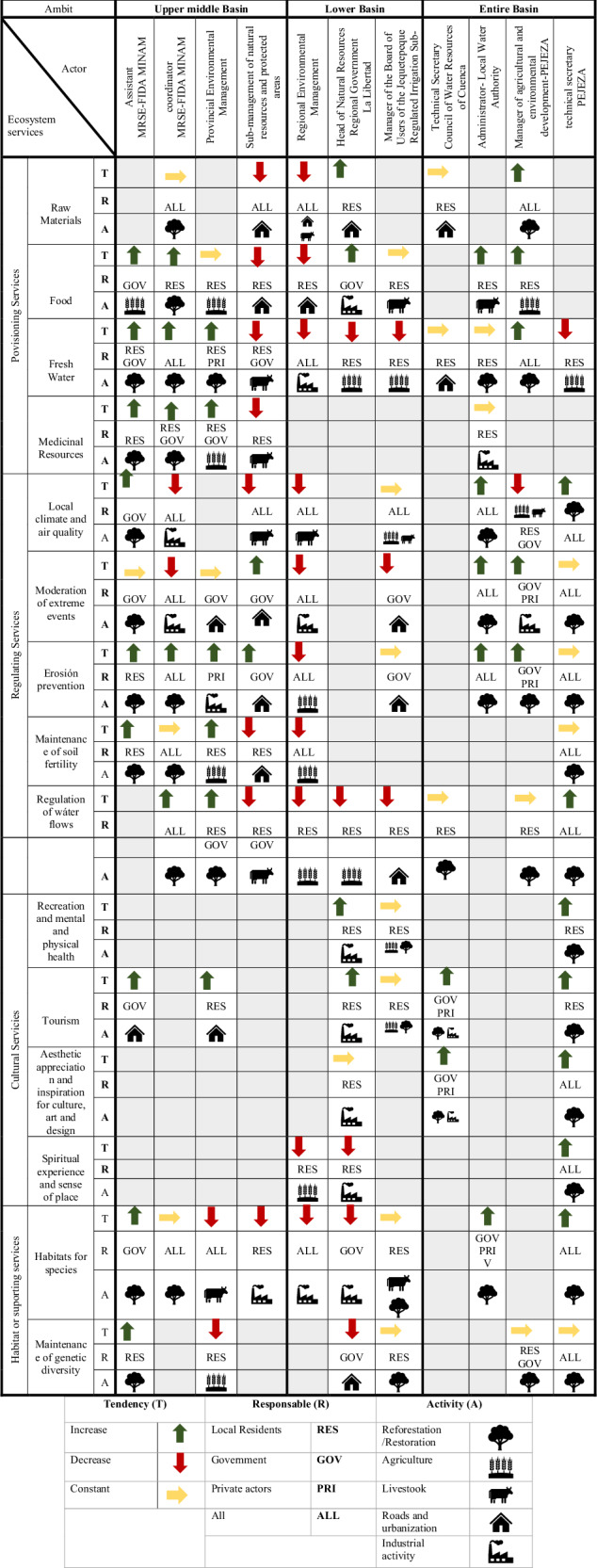
Change value of ecosystem services as perceived by local actors in the Chimulala River Basin (Cajamarca, Peru)

For the change value, actors considered that the provision of ES could either increase, remain constant, or decrease, depending on the specific service and the stakeholder group involved. For instance, institutional actors attributed most changes in ES provision, regardless of their trend (increase, decrease, or stability), primarily to actions taken by local actors (Fig. 7). This includes ES such as food and medicinal resources, recreation, mental and physical health, tourism, and esthetic appreciation and cultural inspiration, which are expected to increase due to expanded activities in agriculture, livestock, restoration, and commercial and industrial development. Similarly, genetic diversity is expected to remain constant due to ongoing reforestation efforts. In contrast, institutional actors attribute the decline in habitat provision for species to industrial and commercial activities, holding all stakeholder groups accountable for this projected decrease.

Unlike the other ES, those related to water provision and regulation revealed differences among institutional actors across management areas (middle-upper basin, lower basin, and the entire basin) (Fig. 7). All actors in the lower basin anticipated a decrease in these ES due to agricultural, industrial, and commercial activities, and to a lesser extent, livestock and road construction associated with urban development. They primarily held residents accountable for this decline (Fig. 7). Similarly, one institutional stakeholder from the middle-upper basin (Cajamarca Regional Government) projected a decrease in water provision and regulation, attributing it to livestock and agricultural activities, and held all actors, except private entities, responsible. Conversely, other institutional actors from the middle-upper basin expected an increase in these ES, attributing it to reforestation activities conducted primarily by residents and regional and district governments. Actors overseeing the entire basin predicted that water provision and regulation ES would either increase or remain constant, again due to reforestation efforts. They considered all actors responsible, with residents mentioned most frequently (Fig. 7).

Regarding local actors, differences emerged in the change value of ES. Local actors from La Arteza and Rodeopampa generally anticipated a decrease in ES, while those from Mutish expected an increase. The decline was primarily attributed to mining activities, as well as the felling and burning of trees associated with livestock activities, whereas the increase was linked to reforestation efforts. Concerning accountability for these changes, local actors across the three communities largely held themselves responsible. However, those from La Arteza and Rodeopampa also attributed the decrease to the State and the private sector. Finally, local actors across all three communities recognized mining as a major driver of change in ES provision, particularly in terms of water supply and air quality.

## Discussion

We propose that the sociocultural valuation of ES could play a critical role in decision-making for IWRM. To this end, we conducted a sociocultural valuation of the ES provided by the ecosystems of the Chimulala micro-watershed. This valuation analyzed the perception, orientation, and change values of various actors to better understand their perceptions, preferences, and motivations that influence the supply and delivery of ES, including those related to water.

### Sociocultural ecosystem services values

Perceptions of ES vary due to a complex array of factors, such as gender and the spatial distribution of sociocultural values associated with ES (Castro et al. 2016; McNally et al. 2016). Martin-López et al. (2012) found that men are more likely to recognize provisioning services, whereas women are inclined to identify regulatory services—a trend aligned with previous studies indicating that women generally display more pro-environmental behaviors than men (Zelezny et al. 2000). This pattern is consistent with our findings among institutional actors, who were predominantly male and identified more provisioning services, whereas women identified a broader array of services. However, this trend does not apply to local actors, as both men and women identified all ES.

Furthermore, our results mirror those of Martin-López et al. (2012), who identified a global tendency for individuals to first acknowledge the importance of provisioning services, followed by regulatory and cultural services. Our study shows variations in the identification, perception, and orientation values of ES among actors, influenced by their proximity to the location. Institutional actors identified and valued fewer ES compared to local actors; similarly, local actors in the lower micro-watershed identified and valued fewer ES than those in the middle and upper regions. These findings align with Castro et al. (2016), Cáceres et al. (2015), García-Llorente et al. (2011), and Hein et al. (2006), who associate sociocultural values of ES with individuals’ familiarity with the area, suggesting that distance or limited experience with a place may restrict knowledge or alter perceptions of its natural and cultural values.

Castro et al. (2016), Martin-López et al. (2012), and Yang et al. (2019) suggest that rural residents tend to recognize and value more ES than their urban counterparts, which may be due to the direct and explicit link between rural residents’ well-being and ES. In our study, institutional actors from urban areas identified and valued fewer ES than local actors from rural areas. Another factor influencing ES perception and valuation is individuals’ primary source of income (Castro et al. 2016; McNally et al. 2016). Cáceres et al. (2015) note that subsistence farmers involved in primary production activities tend to identify more ES than farmers and livestock producers focused on generating surplus. Our results support this, as local actors from the Mutish Peasant Community identified more ES than those from Rodeopampa and La Arteza. In Mutish, livestock production is strictly for subsistence, while in Rodeopampa, the dairy company Gloria S.A. purchases milk from local farmers (Escurra 2001). La Arteza combines subsistence and surplus activities.

Arias-Árevalo et al. (2017) observe that local actors ascribe plural values to ES, encompassing instrumental (economic) values (not measured in this study), as well as intrinsic and relational values (motivations: altruistic, biocentric, and selfish), suggesting a holistic worldview. This comprehensive valuation arises from rural populations’ deep connection with ecosystems, shaped by cultural ties and material dependence on natural resources. Our findings align with this perspective, reflecting the diverse valuation of ES by local actors in this study. Conversely, institutional actors tend to ascribe primarily intrinsic values, often associated with unselfish motivation, without integrating these values with other motivational perspectives.

### Synergies and tensions in the sociocultural assessment of SE

Regarding exchange value, Smith et al. (2013), Chan et al. (2012b), and Summers et al. (2012) emphasize that identifying the factors of change affecting ES provision, and thereby human well-being, is crucial for informed decision-making. This study reveals both synergies and tensions among the change factors influencing ES provision as identified by actors in the micro-watershed. All actors recognized reforestation as an activity likely to enhance the provision of most ES, and most attributed responsibility for ES decline to local actors. While local actors acknowledged their role in ES reduction, they also indicated shared responsibility with institutional actors. These findings align with those of Cano and Haller (2018), who observed that local actors recognize their accountability in ES reduction but also attribute responsibility to institutional actors, while the latter predominantly place the onus on local actors.

Iniesta-Arandia et al. (2014) suggest that direct drivers of change—factors directly influencing ecosystem processes—are more frequently perceived by local actors, whereas decision-makers are more attuned to indirect drivers that alter these direct influences. This study, however, contrasts with this pattern; institutional actors (decision-makers) identify direct change drivers, specifically agricultural and livestock activities, while local actors identify both direct and indirect factors, such as mining, agriculture, and cattle raising. Local actors’ stance on mining likely reflects Peruvian public policy on extractive activities, particularly in the Cajamarca region where the Chimulala micro-watershed is located. In Peru, mining has historically fueled the economy, prompting policies that encourage foreign investment in this sector (Kuramoto and Glave 2007; Bebbington and Mark 2008). In Cajamarca, socio-environmental conflicts have long shaped the relationship between residents, the state, and mining companies (Yacoub et al. 2016), making the region notable for social tensions (Saade 2013). Unlike local actors, institutional actors omit mining activities in their assessments, likely due to their roles as public employees and their alignment with state policies (Castro et al. 2016; Ramos et al. 2018). This illustrates the complexities of integrating social and environmental considerations, as Merrey (2008) notes that in water resource management, the essential connections are not only hydrological and ecological but, crucially, political.

### Implications for IWRM in the region

IWRM seeks to establish a framework for sustainable water management that empowers civil society and manages water resources in a way that benefits all actors through a governance platform (GWP 2000). The findings of this study reveal significant differences in the perception and valuation of ES between government representatives (institutional actors) and local actors, potentially compromising IWRM within the micro-watershed. Our results show that provisioning services and water flow regulation services (hydrological ES sensu Brauman 2015) are considered vital or highly important by most actors, regardless of individual characteristics. These findings are consistent with those of Cáceres et al. (2015), McNally et al. (2016), Castro et al. (2016), Ramos et al. (2018), Yang et al. (2019), Ncube et al. (2021), and Garau et al. (2020). However, these authors also reported that all social actors similarly valued other ES (habitat for species, climate regulation, soil fertility, erosion control, among others), contrasting with this study’s findings. The lower recognition or importance given to non-hydrological ES here may be associated with the country’s current water challenges (Bebbington and Mark 2008; Aldo and Coronel 2011). Brauman et al. (2007), Castro et al. (2011), and Quintas-Soriano et al. (2014) suggest that media coverage of water issues may have heightened public awareness on this topic.

Pahl-Wostl et al. (2011) note that, in practice, water management often prioritizes extraction over other ecosystem functions and processes, fostering a technical approach to resource management (Ramos et al. 2018; Pahl-Wostl et al. 2011; Menzel and Teng 2010). Ramos et al. (2018) and Ncube et al. (2021) add that decision-makers typically emphasize water provisioning services, despite IWRM’s goal of integrated resource management. This trend is evident in our findings, where all institutional actors (decision-makers) rated water provisioning as of vital importance but recognized very few additional ecosystem services.

Felipe-Lucia et al. (2015) observe that government institutions may rely less directly on ES yet hold greater control over managing critical ecosystem properties, such as regulatory and habitat services, which support various other ES utilized across the micro-watershed. In this study, institutional actors identified only a limited range of non-hydrological ES, constraining their capacity to manage these essential ecosystem properties and, consequently, to foster synergies and balance trade-offs among ES.

The second guiding principle of IWRM advocates that water use and management should be rooted in participatory engagement among users, planners, and decision-makers at all levels (GWP 2000). The findings of this study reveal fundamental mismatches in the perceptions, motivations, and values assigned to ES among the social actors analyzed, posing challenges to adhering to this principle and thereby limiting IWRM’s objectives. Our results support Pahl-Wostl et al. (2011), Ncube et al. (2021), and Garau et al. (2020), who argue for recognizing additional ecosystem functions and processes and involving local actors in water resource management to acknowledge water’s plural values.

Regarding contributions to IWRM decision-making, this study identified distinct valuation dynamics of ES between local and institutional actors, impacting IWRM implementation within the basin. One major critique of IWRM is its limited ability to account for the dynamic role of social influences in management, such as competition, conflict, perceptions, interests, and participation (Cook and Spray 2012; Mizanur and Varis 2005; Ekka et al. 2020). This study highlights specific factors—conflict, perceptions, and interests—that hinder effective IWRM in the basin. Additionally, the ES approach adopted here underscores the importance of considering the full suite of ES, rather than isolating hydrological services, to achieve a truly comprehensive vision of IWRM, recognizing that water resources stem from interconnected ecological functions linked to supporting services

## Conclusion

Our study provides an initial approximation of the sociocultural valuation of ES by different actors in the Chimulala micro-watershed. The findings reveal significant disparities between institutional and local actors in identifying and valuing ES, with local actors recognizing a greater diversity and attributing more importance to these services. The results indicate that sacred, cultural, historical, and political values, which contribute to human well-being, are often overlooked in decision-making. Furthermore, the study uncovers conflicts between the two groups that hinder inclusive water management. Institutional actors attribute potential changes in ES provision to local activities, whereas local actors, though aware that their actions might negatively impact ES, feel threatened by institutional decisions, particularly those related to mining. Findings also highlight that, while water resources hold vital importance, other ES—such as climate regulation and species habitat—are equally prioritized by local actors and should be considered within the IWRM framework. Achieving the primary objectives of IWRM within the micro-watershed requires aligning and reconciling the diverse perspectives of actors concerning water resources and other ES. This shift in approach could strengthen environmental governance, reduce conflicts, and support the development of more effective policies.

Finally, we recognize an emerging field of research yet to be explored. Future studies could expand on this sociocultural framework to examine how climate-related changes interact with the sociocultural values identified here, especially concerning the provisioning, regulation, and sociocultural functions of ES. Additionally, research could further deepen these sociocultural insights by incorporating multi-agent decision-making methodologies, particularly to explore how stakeholder values might shape or inform structured water management scenarios.

## Supplementary information

Supplementary Information

## Footnotes

1 National Program of Direct Support to the Poorest

2 Parent association

3 Social program created by Ley No. 24059 and complemented by Ley No. 27470, to offer a daily ration of food to the population considered vulnerable, for helping them to overcome the food insecurity in which they find themselves.

4 Non-School Initial Education Program

5 Social organizations, strategic allies of the State, that provide low-cost food to vulnerable populations.

6 Social groups whose objective is to carry out productive activities and services, such as occupational workshops, preparation of breakfasts, lunches and walks, among others
