# Supplementary material for: Integrated Water Resource Management under Ecosystem Services Approach—The Chimulala Micro-watershed, Peru
Source: Environ Manage. 2024 Dec 6;75(4):945–60. doi: 10.1007/s00267-024-02092-z (PMC11965233; doi:10.1007/s00267-024-02092-z)
Supplement: Supplementary file 1 — Supplementary Information [file 267_2024_2092_MOESM1_ESM.pdf]

Supplementary Information (SI)

Article title: Integrated water resource management under ecosystem services approach – The Chimulala micro-watershed, Peru.

Journal Name: Environmental Management

Authors Names:

Luisa Fernanda Cifuentes-Herrera[a]; Luz Piedad Romero-Duque[b*]; Oscar Eduardo Angulo Núñez[c]; Jenny Maritza Trilleras[d.]

[a] Pontificia Universidad Católica de Perú, Av. Universitaria 1801, San Miguel 15088-Perú, cifuentes.luisa@pucp.edu.pe

[b] Universidad de Ciencias Aplicadas y Ambientales. Calle 222 No. 55-37, Bogotá, Colombia, luz.romero@udca.edu.co

[c] Pontificia Universidad Católica de Perú, Av. Universitaria 1801, San Miguel 15088-Perú, oangulo@pucp.edu.pe

[d] Universidad de Ciencias Aplicadas y Ambientales. Calle 222 No. 55-37, Bogotá, Colombia, jennymtm@gmail.com

[*] Corresponding Author luz.romero@udca.edu.co

ANEX 1. Code of Ecosystem Services for Multiple Correspondence Analysis

Code	SE Name
P1	Raw Materials
P2	Fresh water
P3	Medicinal Resources
P4	Food
R6	Local climate and air quality
R7	Moderation of extreme events
R8	Erosión prevention

R9	Maintenance of soil fertility
R10	Regulation of water flows
H11	Habitats for species
H12	Maintenance of genetic diversity
C13	Recreation and mental and physical health
C14	Tourism Tourism
C15	Aesthetic appreciation and inspiration for culture, art and design
C16	Spiritual experience and sense of place
